# Airborne SAR Radiometric Calibration Based on Improved Sliding Window Integral Method

**DOI:** 10.3390/s22010320

**Published:** 2022-01-01

**Authors:** Lu Li, Fengli Zhang, Yun Shao, Qiufang Wei, Qiqi Huang, Yanan Jiao

**Affiliations:** 1 Aerospace Information Research Institute, Chinese Academy of Sciences, Beijing 100101, China; lilu192@mails.ucas.ac.cn (L.L.); shaoyun@aircas.ac.cn (Y.S.); weiqf@aircas.ac.cn (Q.W.); huangqiqi20@mails.ucas.ac.cn (Q.H.); jiaoyanan21@mails.ucas.ac.cn (Y.J.); 2 College of Resources and Environment, University of Chinese Academy of Sciences, Beijing 100049, China; 3 Laboratory of Target Microwave Properties, Deqing Academy of Satellite Applications, Huzhou 313200, China

**Keywords:** high-resolution SAR, Xinzhou 60, radiometric calibration, corner reflector, sliding window

## Abstract

To verify the performance of the high-resolution fully polarimetric synthetic aperture radar (SAR) sensor carried by the Xinzhou 60 remote-sensing aircraft, we used corner reflectors to calibrate the acquired data. The target mechanism in high-resolution SAR images is more complex than it is in low-resolution SAR images, the impact of the point target pointing error on the calibration results is more obvious, and the target echo signal of high-resolution images is more easily affected by speckle noise; thus, more accurate extraction of the point target position and the response energy is required. To solve this problem, this paper introduces image context information and proposes a method to precisely determine the integration region of the corner reflector using sliding windows based on the integral method. The validation indicates that the fully polarimetric SAR sensor on the Xinzhou 60 remote-sensing aircraft can accurately reflect the radiometric characteristics of the ground features and that the integral method can obtain more stable results than the peak method. The sliding window allows the position of the point target to be determined more accurately, and the response energy extracted from the image via the integral method is closer to the theoretical value, which means that the high-resolution SAR system can achieve a higher radiometric calibration accuracy. Additionally, cross-validation reveals that the airborne SAR images have similar quality levels to Sentinel-1A and Gaofen-3 images.

## 1. Introduction

Synthetic aperture radar (SAR) has all-day, all-weather Earth observation capabilities and is not subject to interference from clouds, rain, and other bad weather, which is important for applications with strict timeliness requirements, such as emergency disaster monitoring, agricultural monitoring, land resource surveys, topographic surveys, and marine dynamic environment measurement [1]. Many SAR satellites have been launched, and the resolution of spaceborne SAR satellites is continuously improving. Examples include ERS-1/2, JERS-1, and other spaceborne SAR systems, as well as ESA’s Envisat ASAR with a 10 m resolution, Sentinel-1A with a 5 m resolution, China’s HJ-1C with a 5 m resolution, and other SAR satellites, particularly Italy’s COSMO-SkyMed, Canada’s Radarsat-2, Germany’s TerraSAR-X, and China’s Gaofen-3 with a 1 m resolution. With the increasing resolution of SAR satellites, some applications, such as target detection and target classification, have required the quantification of SAR data [2]. SAR external calibration is the prerequisite for the application of SAR data quantification. There are two types of external calibration methods: one uses point targets, and the other uses distributed targets. In practice, point targets are easier to achieve than large-scale distributed targets with known scattering characteristics and homogeneous stability, and they can achieve the calibration accuracy required by the design [3]. Therefore, the point target calibration method is often used to calibrate airborne SAR systems [4]. Additionally, point targets can be applied to the SAR images after calibration to ensure the accuracy of the characteristic backscatter coefficients [5].

Corner reflectors are ground reference instruments that are often used for airborne SAR radiometric calibration. They have a simple structure, stable performance, convenient installation, and low cost, satisfying the requirements of a high radar cross-section (RCS) [6]. In particular, the triangular trihedral corner reflector has a wide beamwidth (~20°) in both the azimuth and elevation dimensions [7,8], and is perhaps the most practical instrument for the calibration of cross-frequency SAR systems [7]. In research on calibration algorithms based on corner reflectors, two different methods—the peak method and the integral method—are often used to combine the RCS and response energy of a point target. Gary et al. reported that after the background clutter and noise are corrected, a straightforward numerical integration of the pixel powers for the corner reflector indicates the best and simplest representation of the RCS. They proposed a simple expression for the relationship between the pixel power in the clutter region and the backscatter coefficient by using the integrated energy for the corner reflector and its RCS [3]. Feng et al. calculated the backscatter coefficients, the system calibration constant, and the system transfer function corresponding to the integral method and the peak method using point targets with known RCSs through actual airborne SAR data and obtained the final images, SCRs, and average backscatter coefficients of typical features to verify the feasibility of using point targets to calibrate the airborne SAR system [4]. Chen et al. conducted a response energy evaluation based on the peak method and the integral method for the corner reflector. They found that both the peak method and the integral method have high calibration accuracies when the image quality is good, and that the integral method can be used first when the image quality is unknown [9]. Zheng et al. found that the integral method has a higher calibration accuracy than the peak method through TerraSAR and airborne SAR calibration experiments based on corner reflectors [10]. In the calculation process, the selection of the center position of the corner reflector is essential for the accurate calculation of the point target response energy. Given the presence of the feature whereby the imaging region of the corner reflector is brighter than the background and occupies many image pixels, Runyi used a region growth algorithm for the automatic extraction of the corner reflector’s center in spaceborne SAR images, which uses the “candidate image pixel block” with the largest gray value as the corner reflector’s center on the basis of controlling the number of image pixels [11]. Zhao combined the grayscale distribution characteristics of the target pixels to improve the accuracy of the measurement to the subpixel level using the interpolation subdivision algorithm and constructed a subpixel method that reflects the relationship between the grayscale position of the image pixels in the target region and the center of mass position of the target pixels to obtain the accurate target pixel position [12].

To verify the radiometric performance of the high-resolution fully polarimetric SAR system carried by the Xinzhou 60 remote-sensing aircraft, we use the peak method and the integral method to calibrate the actual airborne remote-sensing data using the corner reflectors, and we evaluate the radiometric quality of the airborne SAR images with regard to the SCR, response energy and calibration constants, and accuracy. The SAR system is based on a coherent imaging mechanism [13], and the random interference of ground elements will produce speckle noise, which will not only degrade the image information, affect the visual effect of the image, and interfere with feature classification, target identification, and detection, but also affect the application of SAR image quantification [14]. Furthermore, the background scattered echoes at the deployment position that do not participate in the calculation of the response energy and calibration constants present more detailed information; therefore, the background image is not necessarily completely homogeneous. Those echoes are also superimposed on the point target’s echoes, affecting the detection of the point target’s response energy, and these echoes are regarded as background clutter. In addition, the current ground reference equipment consists mostly of corner reflectors, which require manual work for angle adjustment, which in turn introduces alignment errors between the radar beam and the normal direction of the corner reflector [12]. In other words, the response energy of the point target is the superposition of the echo energy of the point target, background clutter, speckle noise, and other types of echoes [3]. Therefore, the accurate selection of the point target is a key step in ensuring the effectiveness of SAR radiometric calibration. This paper introduces image context information and proposes a method to precisely determine the integration region of the corner reflector using the improved sliding window integral method. We also verified the radiometric calibration effect of the improved method using the Xinzhou airborne SAR data. We cross-validated the airborne SAR images using Sentinel-1A and Gaofen-3 images acquired at approximately the same time to check the quality of the airborne SAR images calibrated using the improved integral method in a large-scale range.

## 2. Study Area and Data

### 2.1. Experimental Area

**Figure 1:**
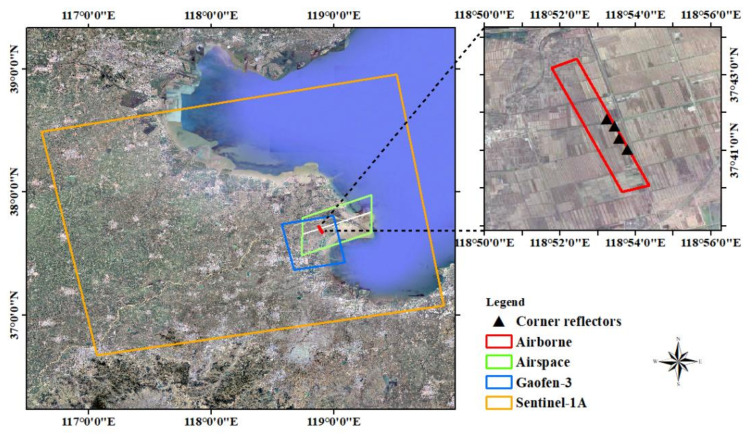
Shandong Dongying comprehensive experimental scope. The orange, blue, and red lines represent the coverage areas of Sentinel-1A, Gaofen 3, and airborne SAR, respectively. The green line indicates the airborne SAR measurement airspace range, and the black triangles represent the corner reflectors.

With the support of China’s National Civil Space Infrastructure Land Observation Satellite Common Application Support Platform Project, the Aerospace Information Research Institute of the Chinese Academy of Sciences conducted a comprehensive synergistic experiment involving the acquisition of spaceborne, airborne, and ground remote sensing data in Dongying, Shandong Province, from 10–25 November 2019. We used the Xinzhou 60 remote-sensing aircraft to acquire P-, L-, C-, and X-band high-resolution SAR data and performed simultaneous ground data acquisition. Dongying is located in the northeastern part of Shandong Province, in the delta of the Yellow River estuary, and its central location is approximately 37.69° N, 118.89° E. The main types of surface coverage are farmland, wetland, waterbody, town, and bare land, and vegetation cover was mainly winter wheat and saline meadow during the experiment. Figure 1 shows the range of the comprehensive experiment in Dongying and the coverage of the multiplatform SAR images. The orange line indicates the coverage area of Sentinel-1A, the blue line indicates the coverage area of Gaofen-3, the green line indicates the airspace area of the Xinzhou 60 remote-sensing aircraft, and the red line indicates the coverage area of the airborne SAR data used in this study. The black triangles indicate the locations of the corner reflectors.

**Figure 2:**
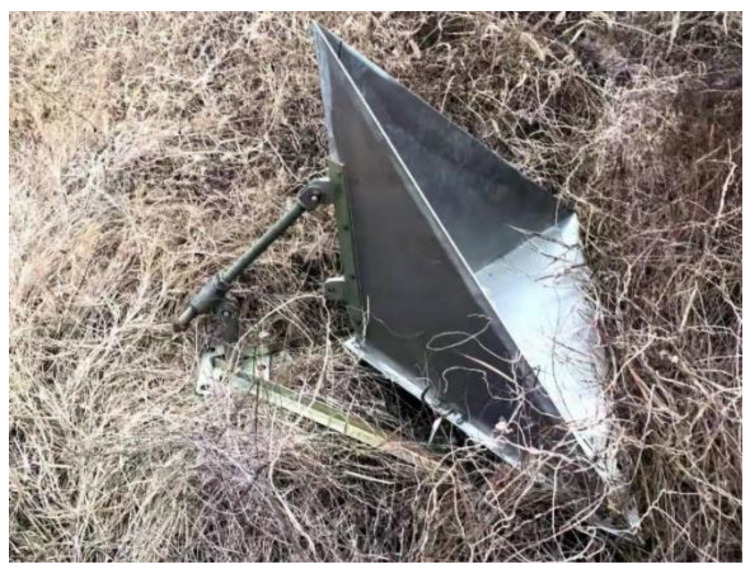
Triangular trihedral corner reflector used in the experiment.

The triangular trihedral corner reflector is perhaps the most practical reference for calibrating SAR systems [8]. The peak RCS value for an ideal trihedral is given as [7]:

(1) $$\sigma_{ref_{i}}=\frac{4\pi a^{4}}{3\lambda^{2}},$$

where $\sigma_{ref_{i}}$ represents the peak value for the RCS of the corner reflector, a represents the leg length of the trihedral, and λ represents the wavelength of the SAR load. As shown in Figure 2, the leg length of the triangular trihedral corner reflector in the experiment was 700 mm, and the peak RCS under the C-band SAR system was approximately 326.309 m^2^, i.e., 25.136 dBsm. In addition, the accuracy of the corner reflector RCS that we used is 0.2 dB.

### 2.2. Airborne SAR Data

During the comprehensive experiment, the Xinzhou 60 remote-sensing aircraft—a high-performance, advanced remote-sensing aircraft independently developed by China—was used to carry the multi-band SAR sensor of the Aerospace Information Research Institute of the Chinese Academy of Sciences [15] to conduct a large-area synergetic flight observation experiment in Dongying and the surrounding area on 25 November 2019. High-resolution SAR data of the P-, L-, C-, and X-bands were obtained, which can be used in topographic surveying and mapping, environmental protection, disaster monitoring, urban planning, scientific research, national security, etc. The parameters of the sensor are presented in Table 1.

**Figure 3:**
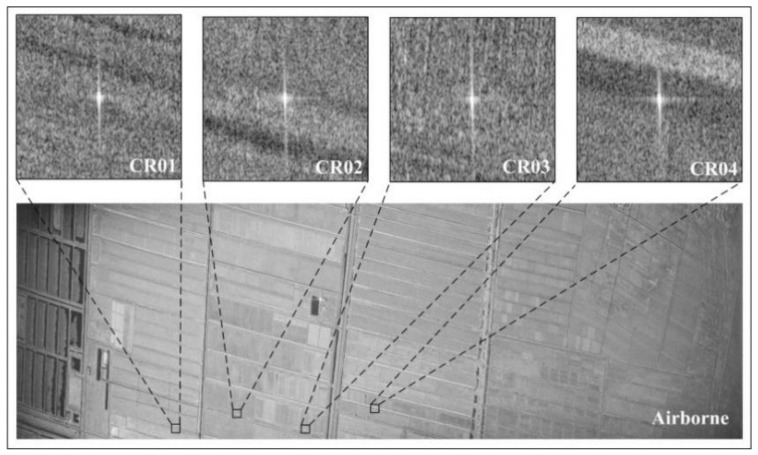
C-band VV polarimetric SAR image and the trihedral corner reflector distribution.

To evaluate the performance of the multi-band high-resolution SAR sensor carried by the Xinzhou 60 remote-sensing aircraft, the C-band was taken as an example, and we selected L1-level single-look complex (SLC) images with full polarization, a resolution of 0.5 m, and a flight altitude of 4500 m, which were processed with POS motion compensation, range compression, azimuth focus, and antenna pattern correction. The specific parameters are presented in Table 2. In the airborne simultaneous observation experiment, a total of four triangular trihedral corner reflectors with leg lengths of 700 mm were placed along the range direction in a uniform farm field in the experimental area, as shown in Figure 3. Because the multi-platform SAR data used for cross-validation all contained VV polarimetric images, the distribution and a local magnification of the corner reflector in the airborne VV polarimetric images are shown in Figure 1 and Figure 3.

### 2.3. Spaceborne SAR Data

To verify the radiometric calibration results of the high-resolution airborne SAR data on a large scale, Gaofen-3 and Sentinel-1A data with an ascending orbit were acquired at approximately the same time as the airborne images, avoiding daily changes of the surface parameter. The specific parameters are presented in Table 3. The original images were all in SLC format, and the image mode was the quad-polarization strip mode for GaoFen-3 and the interferometric wide swath mode for Sentinel-1A. Both airborne SAR and Gaofen-3 acquired fully polarimetric SAR data during the experiment, whereas Sentinel-1A acquired VV&VH dual-polarimetric SAR data. Therefore, the VV polarimetric data of the airborne SAR, Gaofen-3, and Sentinel-1A were used as examples to assess the radiometric calibration quality of the airborne SAR images with regard to both direct and cross-validation. The Gaofen-3 image was acquired on 22 November, with a three-day difference from the time of the airborne SAR synchronization experiment. According to local meteorological data, it could be used considering the lack of rain between 22 and 25 November in the study area. Thus, we selected the backscatter coefficients of stable features in spaceborne SAR images for the cross-validation of the airborne SAR data.

## 3. Methods

**Figure 4:**
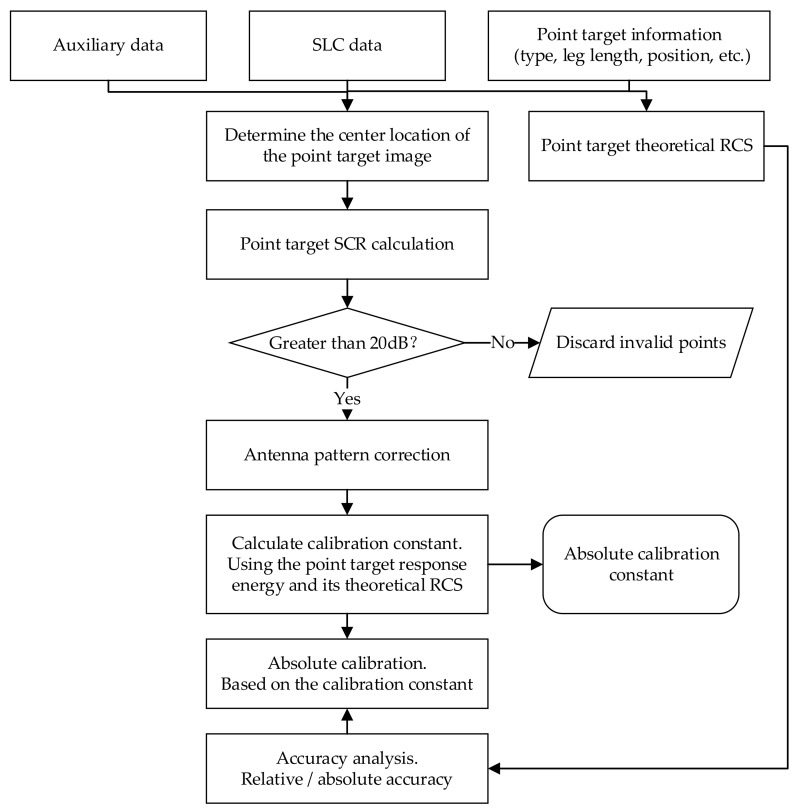
Flowchart of radiometric calibration based on point targets.

SAR radiometric calibration includes measuring the antenna pattern and measuring the overall transfer function of the radar system (or absolute calibration constant) [16]. When the SAR image is absolutely calibrated, it is necessary to calculate the impulse response energy $\varepsilon_{p}$ of the point target and then obtain the absolute calibration constant K (referred to as the calibration constant) of the stable radar system according to $\varepsilon_{p}$ and the RCS σ. Finally, we use the calibration constant to absolutely calibrate the SLC data and evaluate the accuracy of the results. The detailed process of SAR calibration is shown in Figure 4.

### 3.1. Antenna Pattern Correction

Each SAR’s antenna has its antenna pattern. The gain peak is located at the center of the antenna beam and decreases with the increasing angular distance from the center of the beam [17]. The measured power intensity changes according to the beam incidence angle, and when reflected on the SAR image, it manifests as uneven brightness caused by the difference in the incidence angle. Therefore, before the quantitative application of the data, antenna pattern correction is needed to eliminate the amplitude deviation in the range direction of the image caused by the different incidence angles [18]. The basic process of antenna pattern correction based on the corner reflectors is as follows.

(a) Layout corner reflectors: arrange several trihedral corner reflectors of the same leg length at equal intervals along the range direction, accurately determine the coordinates of the corner reflector positions, and adjust the azimuth and elevation angles required for operation.
(b) Antenna pattern fitting: calculate the response energy of each corner reflector in the SAR image and reconstruct the antenna pattern.
(c) Correction coefficient calculation: calculate the ratio of the peak value on the range direction antenna pattern fitting curve to the fitted value of each pixel as the correction coefficient for each position along the range direction.
(d) Image correction: multiply each pixel in the image range direction by the corresponding correction coefficient to eliminate the radiometric deviation caused by the unknown antenna pattern.

### 3.2. Point Target Impulse Response Energy Calculation

**Figure 5:**
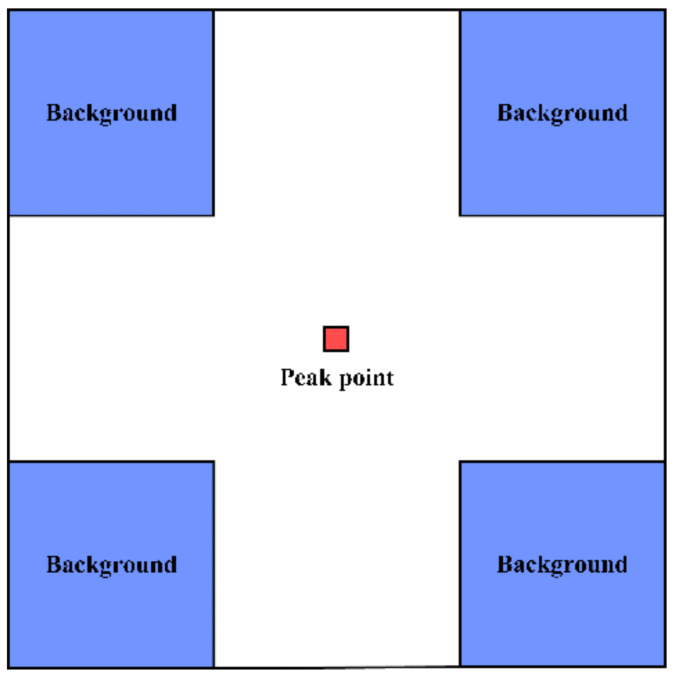
SCR calculation region. The red pixel represents the peak point, and the blue pixels represent the clutter regions.

When SAR images need to be absolutely calibrated, the key to the determination of the calibration constant is accurately extracting the impulse response energy of the point target. The peak method and the integral method are often used to calculate the impulse response energy of the corner reflector [3,9]. To ensure the validity of the calculation results, it is necessary to measure the SCR of the point target in the actual SAR image before calculating the response energy [7], as indicated by Equation (2). The ratio of the peak power in the target impulse response to the average background clutter power is often used as the SCR, which can be estimated using the area near the point target. Figure 5 shows the specific calculation region for the SCR. When the SCR of the point target is greater than 20 dB, the point target can be considered valid for the subsequent calculation of the response energy and other related calculations [19].

(2) $$SCR=\frac{\sigma_{pq}^{T}}{\langle \sigma_{pq}^{C}\rangle }=\frac{\sigma_{pq}^{T}\sin\theta_{i}}{\langle \sigma_{pq}^{o}\rangle \delta_{a}\delta_{r}},$$

Here, $\sigma_{pq}^{T}$ represents the point target RCS, $\langle \sigma_{pq}^{C}\rangle$ represents the average background clutter RCS, $\theta_{i}$ represents the local incidence angle of the point target, and $\delta_{a}$ and $\delta_{r}$ represent the SAR image pixel dimensions in the azimuth and range directions, respectively.

A. Peak estimation method

The peak method is a function of the peak impulse response of the point target and the equivalent resolution cell area. The use of the peak method requires knowledge of the resolution unit of the SAR system [20]; that is, the 3 dB antenna main lobe width of the impulse response. Additionally, it is influenced by the system focus. The formula for calculating the impulse response energy of the peak method based on the point target is [21]:

(3) $$\varepsilon_{r}=DN_{p}^{2}ar\delta_{a}\delta_{r},$$

where $DN_{p}^{2}$ represents the pixel intensity value of the point target, and a and r are the antenna 3 dB IRW (impulse response width) in the azimuth and range direction of the spread, respectively. In the weighted case, the fair width of the IRW is approximately 20%, namely 1.2 to 1.5 pixels [4,22].

B. Integral method

**Figure 6:**
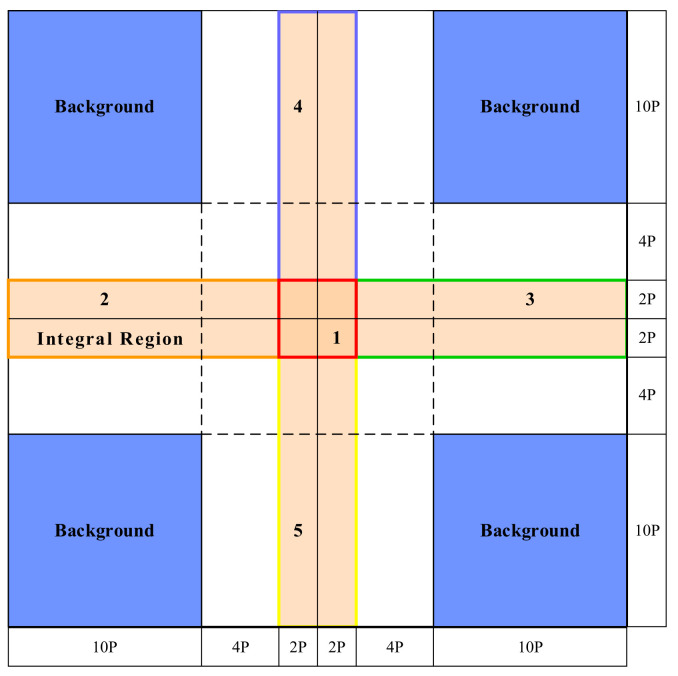
Schematic of the point target response integration area.

The integral method obtains the impulse response energy by integrating the point target impulse response in a specific imaging region. Because the integral method does not need to specify the impulse response of the point target, the calculation is not affected by the system gain, focus, scene, partial coherence of the SAR processor, etc. [3,20]. This feature of the integral method is an advantage over the peak method, and Gary [3] and Cumming [23] have described this idea in detail. In the case of unknown image quality, the integral method is the preferred method for airborne SAR data processing [24]. So far, there is a lot of literature based on the general framework of the integral method and on improving the calibration method for some specific problems, such as the combination of the fit of the sinc antenna pattern and the integral method [18]. The integral response energy of a point target is equal to the difference between the energy of the cross-shaped integration region and the energy of the background calculation region in the integration window of Figure 6 [3].

(4) $$\varepsilon_{p}=\left(\sum_{i\in A}^{N_{A}}DN_{i}^{2}-\frac{N_{A}}{N_{B}}\sum_{i\in B}^{N_{B}}DN_{i}^{2}\right)\delta_{a}\delta_{r},$$

Here, $DN_{i}^{2}$ represents the intensity value of the point target pixel, A represents the point target energy integration region (the number of corresponding image pixels is $N_{A}$), and B represents the background region (the number of corresponding image pixels is $N_{B}$).

The key to the use of the integral method is to accurately and efficiently select the point target integration window to achieve star–ground matching. Therefore, the optimization of the integral method in this paper is for the star–ground matching part of the integration window selection. For the selection of the integration area, the entire point target energy response range must be included, and the background must be relatively uniform to reduce the interference caused by background clutter. The position of the point target is taken as the center, and the $2^{K}\times 2^{K}$(a natural number K that is greater than 2 is selected according to the actual situation; for our experiment, K=5) rectangular area is taken as the integration window. In Figure 6, P represents the size of a pixel, area 1 (red area in the figure) is the main lobe area, area 2 (orange) and area 3 (green) are the azimuth side lobe areas, and area 4 (purple) and area 5 (blue) are the range sidelobe areas.

### 3.3. Optimal Selection of Center Point

**Figure 7:**
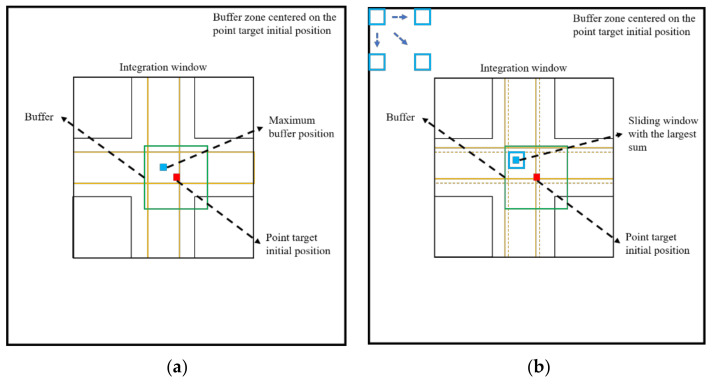
Schematic of the point target initial positioning method and integration window selection based on (**a**) the maximum center method and (**b**) the sliding window method.

As shown in Figure 6, the determination of the point target’s center position is important for accurately selecting the response energy integration window. In an ideal SAR image, the corner reflector appears as a cross-shaped bright spot, and the pixel value is the largest at the spot center, which is the phase center. Therefore, in the traditional method, the maximum center method is often used to determine the center position of the integration area. According to the measured longitude and latitude coordinates of the point target, the initial position on the SAR image is determined by an affine transformation, as indicated by the red pixel in Figure 7. Taking into account the inherent position deviation in the SAR imaging process, a buffer (green line) is set at the initial position of the point target, and the maximum point of the buffer is selected as the center position of the integration window (as indicated by the blue pixel in Figure 7a). However, for the radiometric calibration process of the high-resolution SAR system, the selection of the point target location will inevitably be affected by errors, such as speckle noise [13], background clutter, and alignment errors [12]. This may increase the extraction error of the point target position, affecting the calculation of the integral response energy and the calibration effect. To overcome the difficulties of the maximum center method, we propose the sliding window method to precisely determine the point target location. As shown in Figure 7b, a buffer area (green line) is taken at the initial position of the point target (red pixel), and then a fixed-sized sliding window (blue box) is applied to slide at a speed of one step, and the sum of the DN values of the window is calculated. Finally, the center point of the sliding window with the largest sum is set as the center point of the integration window (blue pixel in Figure 7b). The calculation process is presented in detail in Algorithm 1. For an ideal point target, the point target integration window obtained via the sliding window method is consistent with that obtained via the maximum center method. In the case of deviations, the sliding window method introduces image context information and uses regional statistics to replace the traditional pixel-based positioning method; thus, it can better correct the point target center selection error. In this way, the cross-shaped energy integration area can be corrected to obtain a more accurate response energy integration window (as indicated by the yellow line in Figure 7b).
**Algorithm 1:** Sliding window method point target center location extraction process.Input:  DN: Image amplitude value  Coor_plan: Point target initial position X coordinate  Size_wind: Buffer size  Size_wind: Sliding window size Output:  Coor _corr: Coordinates of the point target position corrected using the sliding window method Process:  DN_buff = Buffer (DN, Coor_plan, Size_wind)  // Buffer Get point target location buffer  Sum_wind = 0  **for** i = 0 to Size_buff-Size_wind do   **for** j = 0 to Size_buff-Size_wind do    window = Window(i, j, Size_wind)   // Window Get the window corresponding to position i, j    **if** Sum(window)> Sum_wind then   // Sum Calculate the sum of pixels in the window     Sum_wind = Sum(window)     Corr_buff = i, j    **end if**   **end for**  **end for**  Coor _corr = Coor_plan-Size_wind/2 + Corr_buff

### 3.4. Calibration Constant Calculation

When SAR processors need to be absolutely calibrated, it is often necessary to deploy several corner reflectors on the ground to cooperate with the airborne or spaceborne SAR for simultaneous observation. The peak or integral method is then used to extract the corner reflector impulse response energy values and obtain the calibration constants in combination with the known RCS of the corner reflector and its local incidence angle. Specifically, N corner reflectors are placed on the ground, the impulse response energy of corner reflector i is denoted as $\varepsilon_{p_{i}}$, the reference RCS is denoted as $\sigma_{ref_{i}}$, and the local incidence angle is denoted as $\theta_{i}$; then, the calibration constant $K_{i}$ of corner reflector i is given as [18,25]:

(5) $$K_{i}=\frac{\varepsilon_{p_{i}}\sin\theta_{i}}{\sigma_{ref_{i}}}.$$

The average method [26] is often used, which involves taking the average value of the calibration constants of all corner reflectors as the whole scene image’s final calibration constant for improving the calculation accuracy [4,26]:

(6) $$\bar{K}=\frac{1}{N}\sum_{i=1}^{N}K_{i},$$

After the average value of the calibration constant $\bar{K}$ is obtained, Equation (7) is often used to calculate the backscatter coefficient [4]:

(7) $$\sigma_{0}=\frac{DN^{2}}{\bar{K}}\cdot \sin\theta ,$$

here, $\sigma_{0}$ represents the value of the backscatter coefficient corresponding to the feature, and $DN^{2}$ represents the intensity value of the point target pixel. For SLC data, $DN^{2}$ is calculated using the real part I and imaginary part Q; i.e., $DN^{2}=I^{2}+Q^{2}$.

### 3.5. Radiometric Calibration Accuracy Analysis

Radiometric calibration can correct the radiometric distortion caused by the expansion loss effect, non-uniform antenna patterns, gain changes, and speckle noise during SAR imaging processing [17], but the system gain, integrated sidelobe response, etc., may lead to calibration errors. The relative calibration accuracy and absolute calibration accuracy are often used as evaluation indices to describe the accuracy and stability of SAR images.

A. Relative calibration accuracy

Corner reflectors with an equal theoretical RCS should have the same response energy in the SAR image after antenna pattern correction. A certain number of corner reflectors with the same theoretical RCS are placed in the calibration field, and the measured RCS of each corner reflector is calculated and statistically analyzed to obtain the relative calibration accuracy.

(8) $$\Delta R=\sqrt{\sum_{i=1}^{N}\frac{{\left(\hat{RCS_{i}}-\bar{RCS_{a}}\right)}^{2}}{N}},$$

Here, ΔR represents the relative calibration accuracy, $\hat{RCS_{i}}$ represents the measured RCS value of the corner reflector i, and ${\bar{RCS}}_{a}$ represents the average measured RCS value for the N corner reflectors.

B. Absolute calibration accuracy

Determining the absolute calibration accuracy involves using the calibration constant to calibrate the impulse response of corner reflectors with a known RCS in SAR images to obtain the measured RCS after calibration. The absolute value of the difference between the measured RCS of each corner reflector and the corresponding theoretical RCS is obtained, and the maximum value of the absolute values is taken as the absolute calibration accuracy of the SAR image [18].

(9) $$\Delta A=Max\left|{\hat{RCS}}_{i}-RCS_{i}\right|,$$

Here, ΔA represents the absolute calibration accuracy, and $RCS_{i}$ represents the theoretical RCS value of the corner reflector i. With a reduction in ΔA, the difference between the measured and theoretical values is smaller, the absolute calibration accuracy is higher, and the image characteristics are better.

## 4. Results and Discussion

### 4.1. Antenna Pattern Correction Evaluation

**Figure 8:**
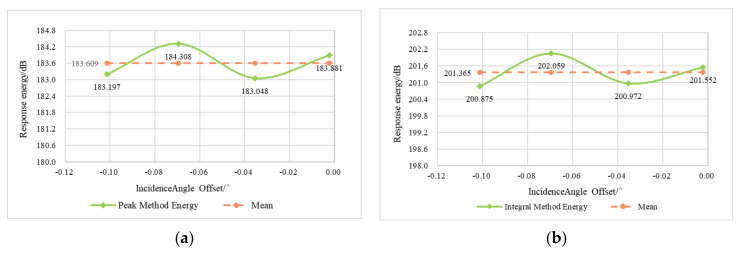
Verification of the correction results for the corner reflector antenna pattern in the VV polarimetric image: (**a**) peak method; (**b**) integral method.

To verify the correction effect of the antenna pattern, the VV polarimetric data were used as an example, and the peak method and the integral method were used to extract the response energy of the corner reflector, as shown in Table 4. The mean energy value obtained via the peak method was 183.609 dB (with a standard deviation of 0.591), and the mean energy value obtained via the integral method was 201.365 dB (with a standard deviation of 0.551). The results indicate that the dispersion and fluctuation of the energy values of the corner reflector are small. As shown in Figure 8, there is no overall tendency for the corner reflector energy to exhibit a significant change in magnitude in the range direction due to the difference in incidence angle offset. Instead, it fluctuates slightly around the mean, indicating that the data are relatively well corrected for the antenna pattern.

### 4.2. Comparative Analysis of Peak Method and Integral Method

#### 4.2.1. Point Target SCR Analysis

It is necessary to ensure that the imaging of the point target in the SAR image is affected by the background clutter as little as possible; that is, the SCR of the corner reflector SCR is required to be greater than 20 dB. The VV polarimetric data were also used as an example to verify the SCR results with the corner reflectors. As shown in Table 5, the SCR values of all corner reflectors are greater than 35 dB, indicating that each corner reflector can be considered to be a valid point target in the subsequent calculation.

#### 4.2.2. Calibration Results and Accuracy Analysis

**Figure 9:**
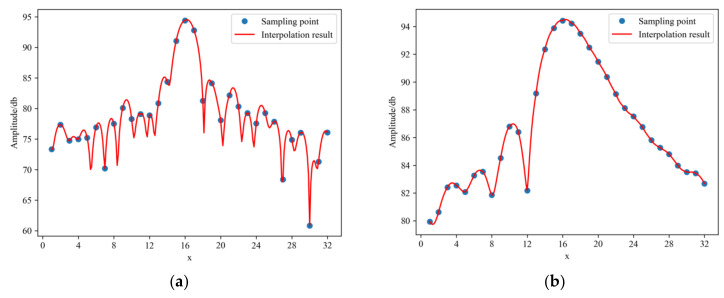
CR03 one-dimensional profile of the impulse response in the range and azimuth directions in the VV polarimetric image: (**a**) range direction interpolation; (**b**) azimuth direction interpolation.

The peak response of the corner reflector occupies only one or two pixels in the SAR image, and a fine description of the corner reflector impulse response through interpolation is required. We verified the results of four, eight, and sixteen fast Fourier transform (FFT) interpolations and found that eight-fold FFT interpolation can achieve sufficient accuracy with guaranteed computational efficiency. Therefore, an eight-fold FFT interpolation was performed in a 32 × 32 neighborhood of the phase center of the corner reflector. The profile lines of the response function in the range and azimuth directions are displayed in a one-dimensional form, as shown in Figure 9.

**Figure 10:**
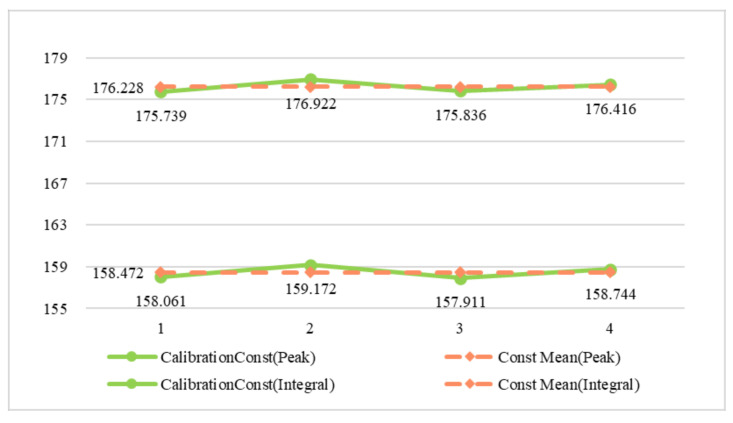
Comparison of the calibration constants and mean values for the VV polarimetric image.

The response energy and calibration constants of each corner reflector are calculated using the peak response and integration region obtained by interpolation. Theoretically, the calibration constants of each corner reflector used in the experiment should be the same in the SAR image after antenna pattern correction. The stability of the results of the two response energy calculation methods is verified by comparing the standard deviations of the calibration constants of the corner reflectors obtained by the two methods. As shown in Table 6, the mean value of the response energy for the peak method is 183.609 dB, and the mean value of the calibration constant obtained by using the theoretical RCS value is 158.472 dB (with a standard deviation of 0.591). The mean value of the integral response energy of the four corner reflectors determined by the integral method is 201.365 dB and the mean value of the calibration constant is 176.228 dB (with a standard deviation of 0.551). In Figure 10, the calibration constants (green line) corresponding to the peak method exhibit larger fluctuations than the mean value (red line), while the calibration constants (green line) obtained via the integral method exhibit smaller fluctuations than the mean value (red line). The differences between the CR03 calibration constants obtained via the peak and integral methods and their corresponding mean values are 0.561 and 0.392 dB, respectively. In summary, the standard deviation of the calibration constants obtained via the integral method is smaller than that for the peak method, indicating that the calculation results obtained via the integral method are more stable.

According to the principle of radiometric calibration and accuracy evaluation, the average values of the calibration constants calculated via the peak method and the integral method are used as the calibration constant of the whole image for the absolute calibration and the accuracy evaluation of SAR images, respectively. As shown in Table 7, the relative calibration accuracy of the integral method is 0.551 dB and the absolute calibration accuracy is 0.668 dB. For the peak method, the relative calibration accuracy is 0.591 dB and the absolute calibration accuracy is 0.67 dB. The results indicate that the calibration accuracy of the integral method is better than that of the peak method.

### 4.3. Improved Method and Results Based on Sliding Windows

#### 4.3.1. Center Point Optimization Analysis

To further improve the calibration accuracy, the positioning of the center point of the corner reflector is optimized by sliding the window, and the SCR of the corner reflector is calculated via the traditional maximum center method and the sliding window method. As shown in Table 8, the image coordinates of CR01 and CR03 differ by one image element in the X direction (azimuth direction), and the image coordinates of CR02 and CR04 are identical. The mean SCR of the maximum center method is 38.126 dB, and the corresponding mean SCR of the sliding window method is 38.130 dB. The mean SCR is improved after optimization, which indicates that the sliding window method can reduce the interference of background clutter.

**Figure 11:**
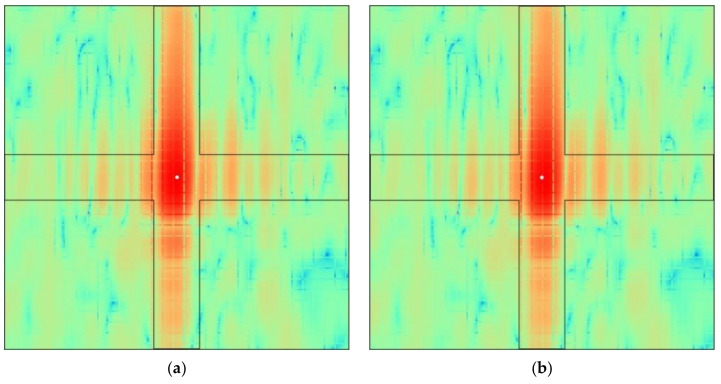
Integral window of corner reflector CR03 before and after center point position optimization: (**a**) maximum center method; (**b**) sliding window method.

The sliding window is used again for center position selection in the integration region at the subpixel level after FFT interpolation. To clearly compare the selection effects of the integration region before and after optimization, the integration window of the corner reflector is visualized with CR03 as an example and the theoretical cross-shaped integration region of the corner reflector is marked with a solid black line (Figure 11). The center points determined using the sliding window method and the maximum center method are indicated by the white dots in Figure 11. As shown, the corner reflector positions determined via the two methods differ by four subpixels in the X-direction and one subpixel in the Y-direction. Statistical analysis of the cross-shaped integration regions obtained through the two methods indicates that the integral energy obtained through the maximum center method was 216.51 dB, and the integral response energy obtained through the sliding window method was 216.53 dB; thus, the result of the maximum center method was 0.02 dB lower than that of the sliding window method. Assuming that 145 dB is the threshold value, the number of pixels in the cross-shaped integration region with values lower than the threshold value obtained via the maximum center method is 972, while the number of pixels in the integration region obtained via the sliding window method is 818. Therefore, the number of pixels with a scattering intensity of <145 dB in the integration region obtained via the sliding window method is 154 less than that of the maximum center method, which accounts for approximately 1% of the total number of pixels in the integration region. The energy integration region determined using the maximum center method contains more background pixels; thus, the calculated integral response energy value is small. However, the integration window obtained via the sliding window method can significantly reduce the number of background pixels, which makes the integral response energy extracted from the SAR image closer to the theoretical value of the corner reflector and improves the calculation accuracy.

#### 4.3.2. Calibration Results and Accuracy Analysis

The center point of the integration region is extracted via the methods mentioned above, the response energy of the point target is extracted using the integral method, and the corresponding calibration constants are calculated. As shown in Table 9, the average value of the integral response energy of the point target obtained via the maximum center method is 201.365 dB, and the mean value of the calibration constant calculated by using the theoretical RCS value is 176.228 dB (with a standard deviation of 0.551). The average value of the response energy for the integration regions of the four point targets obtained via the sliding window method is 201.379 dB, and the average value of the calibration constant is 176.242 dB (with a standard deviation of 0.546). When the point target position coordinates, response energy, and calibration constant are employed, the response energy of the corner reflector obtained via the sliding window method is more concentrated than that obtained via the maximum center method. Additionally, the standard deviation of the calibration constants is better for the sliding window method than for the maximum center method, indicating that the integral method improved by the sliding window is more stable and more robust.

The average value of the calibration constants obtained via the two integral methods is used as the calibration constant for the entire SAR image to calibrate the SLC data. The measured RCS values of each corner reflector are extracted from the calibration image, and the calibration accuracy is compared with the theoretical RCS value. The relative calibration accuracy and absolute calibration accuracy are used as indicators to evaluate the effect of radiometric calibration before and after optimization. As shown in Table 8 and Table 10, and Figure 11, the actual integration regions of the four corner reflectors obtained by the sliding window method are significantly corrected, the actual cross-shaped energy integration region overlaps more with the theoretical energy integration region, and the RCS of each corner reflector is improved compared to that of the maximum center method. For the sliding window method, the absolute calibration accuracy is 0.664 dB, and the relative calibration accuracy is 0.546 dB. For the maximum center method, the absolute calibration accuracy is 0.668 dB, and the relative calibration accuracy is 0.551 dB. The calibration accuracies obtained via the sliding window method and the maximum center method both satisfy the radiometric accuracy requirements of the various applications proposed by Ulaby [7] in his report on the scientific requirements for SAR calibration. The relative and absolute calibration accuracies of the improved sliding window integral response energy are better than those obtained via the maximum center method, and the optimized method has more stable and robust calculation results, indicating that the optimized sliding window integral method can significantly improve the quality of airborne SAR calibration.

### 4.4. Cross-Validation Analysis

**Figure 12:**
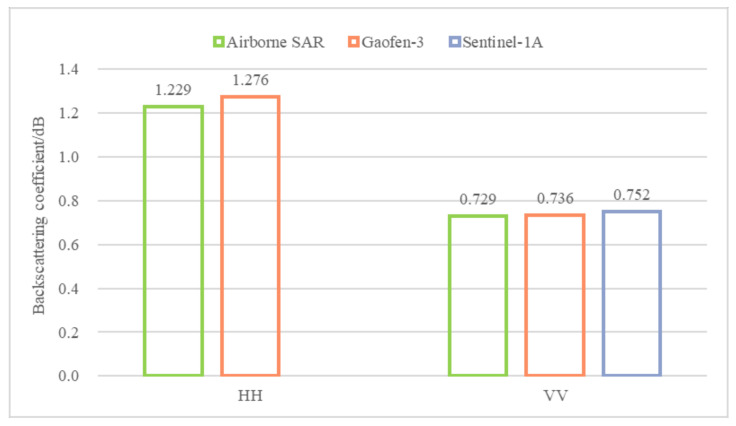
Comparison of normalized backscatter coefficients in the building area obtained with different sensors.

To verify the radiometric calibration results of high-resolution airborne SAR data based on the integral method improved by the sliding window on a large scale, the optimized integral method was applied to the calibration of airborne HH and VV polarimetric images from the experiment, and the Gaofen-3 and Sentinel-1A images acquired at approximately the same time as the airborne SAR data were used for cross-validation. Taking into account the 3-day difference between the Gaofen-3 data and the time of the airborne SAR synchronization experiments, differences in precipitation and wind speed may lead to changes in the intensity of the backscatter of large, distributed features, such as farmlands and ponds. Additionally, the spatial resolution of Sentinel-1A is low, and small areas or independent features, such as roads, are difficult to identify via visual interpretation. To ensure the reliability of the validation results, the cross-validation selects features whose backscatter coefficients do not easily change over time for comparison; that is, the comparison of the backscatter coefficients in the building area. The backscatter coefficients of buildings in different images cannot be directly compared due to differences in the incidence angles of the SAR data from the three platforms. Therefore, according to reference [27], the experiments were performed using a theoretical approach based on Lambert’s law for optics as proposed by Ulaby [28] for the radiometric normalization of the multiplatform data, and the normalized backscatter coefficients with a reference incidence angle of 35° were obtained and compared. As shown in Figure 12 and Table 11, the average value of the backscatter coefficient of the building area for the sensors under HH polarization is 1.25 dB (with a standard deviation of 0.03), while the average value under VV polarization is 0.74 dB (with a standard deviation of 0.01). The backscatter coefficient of the building area under VV polarimetric data is slightly smaller than that under HH polarimetric data, which is consistent with the regular imaging of the fully polarimetric SAR system. The maximum difference between the building area backscatter coefficients of the airborne SAR and spaceborne SAR (Gaofen-3) images in the HH polarimetric data is 0.05 dB, and the maximum difference between those of the airborne SAR and spaceborne SAR (Sentinel-1A) images in the VV polarimetric data is 0.02 dB. In summary, the airborne SAR calibration results are consistent with the backscatter coefficients of the building area in the spaceborne SAR data, indicating that the airborne SAR data have high reliability and can effectively reflect the radiometric characteristics of the ground features.

## 5. Conclusions

We used the peak method and the integral method combined with ground corner reflectors to calibrate airborne SAR data and found that the high-resolution SAR system carried by the Xinzhou 60 remote-sensing aircraft can effectively reflect the radiometric characteristics of the ground features. To address many factors that influence the imaging of the high-resolution SAR system, this paper introduced image context information and proposed a method to precisely determine the integration region of the corner reflector using sliding windows based on the integral method. We used high-resolution airborne SAR data acquired by the Xinzhou 60 remote-sensing aircraft for verification. The results indicated that the sliding window allows the position of the point target on the image to be determined more accurately, and the response energy extracted from the image based on the integral method is closer to the theoretical value of the point target, which enables the high-resolution SAR system to achieve higher calibration accuracy. Additionally, cross-validation of the calibrated airborne SAR images with multi-platform spaceborne SAR images acquired at approximately the same time indicated that the airborne SAR images had a similar quality level to the Sentinel-1A and Gaofen-3 images, and have the data conditions for quantitative research.

## Figures and Tables

**Table 1:** Multi-band fully polarimetric SAR parameters.

Band	P	L	C	X
Center frequency/GHz	0.45	1.25	5.4	9.6
Type of polarization	VV&VH	AHV	AHV	AHV
Range resolution/m	3.0	3.0–5.0	2.0–3.0	0.3–3.0
Azimuth resolution/m	3.0	3.0–5.0	2.0–3.0	2.0–3.0
Height accuracy/m	3–6	—	—	1–3
Geometrical and radiometric accuracy	Can satisfy feature classification requirements			

**Table 2:** C-band microwave loading experiment and SLC image parameters.

Sensor Parameter	C-SAR	Image Parameters	Content
Field of view (FOV)	35–55° Left	Radar frequency/GHz	5.4
Relative altitude/m	4500	Type of polarization	Full polarization
Resolution/m	0.5	Range pixel space/m	0.20
Bandwidth/km	3.3 (Antenna)	Azimuth pixel space/m	0.14
Route Offset/km	3.2	Center incidence angle/°	53.65°

**Table 3:** Gaofen-3 and Sentinel-1A image parameters.

Sensor	Gaofen-3	Sentinel-1A
Imaging Time	22 November 2019 15:52:24	25 November 2019 16:01:48
Band	C	C
Center frequency/GHz	5.400	5.405
Imaging Mode	QPSI	IW
Resolution/m	8	20
Range spacing/m	2.248	2.330
Azimuth spacing/m	4.849	13.932
Type of polarization	Quad	VV&VH
Direction	Ascending	Ascending
Incidence angle/°	25.37~28.02	41.49~45.90

**Table 4:** Validation and analysis of the relative correction of the point target response energy.

Corner Reflector	Peak Method			Integral Method		
	Measured Energy/dB	Mean/dB	Standard Deviation/dB	Measured Energy/dB	Mean/dB	Standard Deviation/dB
CR01	183.197	183.609	0.591	200.875	201.365	0.551
CR02	184.308			202.059		
CR03	183.048			200.972		
CR04	183.881			201.552		

**Table 5:** Coordinates and SCR statistics corresponding to each point target in the VV polarimetric image.

Corner Reflector	Longitude	Latitude	X-Coordinate	Y-Coordinate	SCR/dB
CR01	118.898	37.680	5574	7905	37.768
CR02	118.895	37.685	7680	7332	37.305
CR03	118.893	37.690	10,064	7864	37.175
CR04	118.889	37.695	12,494	7145	40.255
Mean SCR/dB			38.126		

**Table 6:** Point target response energy and calibration constants obtained by the peak method and the integral method for the VV polarimetric image.

Corner Reflector	Length/mm	Theoretical RCS/dBsm	Peak Method		Integral Method	
			Response Energy/dB	Constant/dB	Response Energy/dB	Constant/dB
CR01	700	25.136	183.197	158.061	200.875	175.739
CR02	700	25.136	184.308	159.172	202.059	176.922
CR03	700	25.136	183.048	157.911	200.972	175.836
CR04	700	25.136	183.881	158.744	201.552	176.416
Mean response energy			183.609		201.365	
Mean calibration constants			158.472		176.228	
Standard deviation of calibration constants			0.591		0.551	

**Table 7:** Point target RCS and accuracy analysis based on the peak method and integral method.

Corner Reflector	Theoretical RCS/dBsm	Peak Method		Integral Method	
		Measured RCS/dBsm	Difference/dBsm	Measured RCS/dBsm	Difference/dBsm
CR01	25.136	24.694	−0.442	24.621	−0.515
CR02	25.136	25.806	0.67	25.804	0.668
CR03	25.136	24.545	−0.591	24.717	−0.419
CR04	25.136	25.378	0.242	25.297	0.161
Relative calibration accuracy		0.591		0.551	
Absolute calibration accuracy		0.670		0.668	

**Table 8:** Image coordinates and SCRs of corner reflectors based on the original center position and the improved center position.

Corner Reflector	Longitude	Latitude	Maximum Center Method			Sliding Window Method		
			X	Y	SCR/dB	X	Y	SCR/dB
CR01	118.898	37.680	5574	7905	37.768	5573	7905	37.784
CR02	118.895	37.685	7680	7332	37.305	7680	7332	37.305
CR03	118.893	37.690	10,064	7864	37.175	10,063	7864	37.188
CR04	118.889	37.695	12,494	7145	40.255	12,494	7145	40.255
Mean SCR			38.126			38.130		

**Table 9:** Integral response energy and calibration constants of the point target for the maximum center method and sliding window method.

Corner Reflector	Length/mm	Theoretical RCS/dBsm	Maximum Center Method		Sliding Window Method	
			Response Energy/dB	Constant/dB	Response Energy/dB	Constant/dB
CR01	700	25.136	200.875	175.739	200.894	175.757
CR02	700	25.136	202.059	176.922	202.068	176.932
CR03	700	25.136	200.972	175.836	200.991	175.855
CR04	700	25.136	201.552	176.416	201.561	176.424
Mean response energy			201.365		201.379	
Mean calibration constants			176.228		176.242	
Standard deviation of calibration constants			0.551		0.546	

**Table 10:** Point target RCS and accuracy analysis for the maximum center method and sliding window method.

Corner Reflector	Theoretical RCS/dBsm	Maximum Center Method		Sliding Window Method	
		Measured RCS/dBsm	Difference/dBsm	Measured RCS/dBsm	Difference/dBsm
CR01	25.136	24.621	−0.515	24.624	–0.512
CR02	25.136	25.804	0.668	25.800	0.664
CR03	25.136	24.717	−0.419	24.723	–0.413
CR04	25.136	25.297	0.161	25.293	0.157
Relative calibration accuracy		0.551		0.546	
Absolute calibration accuracy		0.668		0.664	

**Table 11:** Statistical results for the backscatter coefficients in the building area obtained with different sensors.

Sensors	HH Polarization	VV Polarization
Airborne SAR	1.229	0.729
Gaofen-3	1.276	0.736
Sentinel-1A	—	0.752

## Data Availability

Not applicable.
